# Orexins and neuregulins as exemplars of peptide physiology in the brain: experimental challenges and implications for neuropathology

**DOI:** 10.3389/fncel.2026.1933123

**Published:** 2026-09-29

**Authors:** Laura Clara Grandi, Marta Cerina, Alida Amadeo, Andrea Becchetti

**Affiliations:** 1 Department of Biotechnology and Biosciences and NeuroMI, University of Milano-Bicocca, Milan, Italy; 2 Department of Experimental and Clinical Medicine, University of Florence, Florence, Italy; 3 Department of Biosciences, University of Milan, Milan, Italy

**Keywords:** cerebral cortex, diffusion coefficient, ErbB, hypocretin, neuropeptide, neurotrophin, NRG

## Abstract

Neuropeptides and neurotrophic factors exert overlapping roles on synaptic maturation and plasticity. Although the relative intracellular signals are mostly elucidated, difficulties are met in discerning the dynamics of their extracellular action *in vivo*, which is largely determined by their peptide nature. We discuss these common mechanisms in two exemplary systems, which are otherwise biologically divergent. The hypothalamic orexin system has a low molecular complexity (two peptides, two receptors), but an intricate connection pattern covering subcortical and cortical structures. Orexins sustain motivated behavior during active waking and are implicated in narcolepsy with cataplexy, as well as psychiatric and metabolic disorders. Neuregulins control neuronal and synaptic maturation after proteolytic release in the extracellular space, or functioning as transmembrane cell-adhesion proteins. They are implicated in neurodevelopmental pathologies and display a wide molecular diversity, with multiple genes and tens of cell- and compartment-specific isoforms. Understanding how peptides regulate synaptic circuits is hampered by uncertainty about essential quantitative aspects: rate of release and diffusion; distribution and desensitization of receptor subtypes; extracellular matrix binding; kinetics of proteolytic degradation. Ongoing studies investigate the peptide-dependent interplay of different CNS populations, by applying optogenetics and ever more specific pharmacological agents to conditional animal models. These approaches need to be complemented by assessing peptide dynamics with novel amperometry or live imaging to monitor peptide release and spatiotemporal distribution, with photoactivatable drugs for spatiotemporal control of stimulus onset, with mass spectrometry to test the long-term cerebrospinal peptide concentration. Furthermore, experimental settings are needed that better mimic the situation *in vivo*.

## 1 Introduction: peptide-dependent signaling in the nervous system

The prototype of neurotrophic factors, NGF, is released from cells in the target area of extending neurites. Besides exerting neurotropic action, NGF is absorbed by the neuron’s terminals and fosters neuron survival and differentiation (Levi-Montalcini, 1987). The neurotrophic factors akin to NGF were named *neurotrophins*. The mammalian comprise BDNF (brain-derived neurotrophic factor), neurotrophin-3 and neurotrophin-4. Neurotrophins are synthesized as larger pro-neurotrophins, which are mostly cleaved intracellularly to yield the mature peptides, which act as dimers. The secreted peptide/pro-peptide ratio modulates the balance of pro-survival and death signals. Neurotrophins stimulate survival and differentiation by activating tropomyosin-related Tyr-kinase receptors (TrkA-C), generally complexed with p75 neurotrophin receptor. Pro-neurotrophins stimulate neuronal death through high-affinity binding with p75 neurotrophin receptor/sortilin complexes (Bothwell, 2019). Ligand-receptor complexes are internalized and regulate transcription after being transferred to the cell body or activating retrograde signals (Harrington and Ginty, 2013).

Shortly after NGF discovery, hypothalamic peptides regulating pituitary endocrine release were identified (Guillemin, 2005). Subsequently, more than one hundred peptides were identified with distinct neuronal distributions (Ludwig and Leng, 2006). These peptides regulate synaptic transmission at different timescales and are denoted as *neuropeptides*. Most neurons release one or more neuropeptides, which are generally, although not always, co-expressed with classical neurotransmitters. Neuropeptides are structurally heterogeneous, with sequences ranging from 3 to ⁓100 amino acid residues (aa), and act on target cells by activating G-protein-coupled receptors (Salio et al., 2006; van den Pol, 2012).

Despite the structural differences, reflected in unrelated evolutionary histories (Lucaci et al., 2022; Leroy et al., 2026), and the specificity for distinct classes of receptors, neuropeptides and neurotrophic factors present overlapping functions. Neurotrophic factors can also modulate synaptic plasticity, while neuropeptides can stimulate survival and proliferation. For instance, BDNF regulates spike-timing dependent long-term potentiation in the hippocampus (Edelmann et al., 2015; Lin et al., 2018) and amygdala (Li et al., 2011). Conversely, neuropeptide-Y sustains proliferation of olfactory and hippocampal precursor cells (Hansel et al., 2001; Howell et al., 2003). Importantly, because of their peptide nature, neuropeptides and neurotrophic factors share essential mechanisms of action, especially regarding their extracellular dynamics. The following paragraphs describe these commonalities, which present analogous experimental challenges, thus justifying our unified treatment of ‘peptide physiology’.

Classic neurotransmitters are stored in small clear synaptic vesicles clustered near the active presynaptic release sites (Salio et al., 2006). Neuropeptides and neurotrophins are stored in large granular vesicles (LGVs) located at a distance from the specialized presynaptic area (Baginska et al., 2023). Clear vesicle release is triggered by quick local [Ca^2+^]_i_ elevation caused by action potential-dependent opening of voltage-gated calcium channels, possibly supported by Ca^2+^ release from intracellular stores. Instead, LGV release is driven by slower [Ca^2+^]_i_ build-up, determined by burst firing patterns (Bondy et al., 1987; Muschol and Salzberg, 2000; Baginska et al., 2023). Neuropeptides can be also released along the axon (Sobota et al., 2020) and from dendrites (Ludwig and Leng, 2006), where local synthesis may occur (Miroci et al., 2012). In CNS, neuropeptides and neurotrophins reach much lower extracellular concentrations than classic neurotransmitters, as few and small LGVs are found in axon terminals. However, peptide size warrants binding affinities in the low nanomolar range (van den Pol, 2012), compared with EC_50_’s of 10–100 μM for ionotropic glutamate receptors (Traynelis et al., 2010) and nicotinic ACh receptors (Becchetti et al., 2023), or 1–10 μM for GABA_A_ receptors (Farrant and Nusser, 2005).

Upon release, fast neurotransmitters reach saturating (millimolar) concentrations in the synaptic cleft. Next, the quasi-simultaneous activation of densely packed postsynaptic receptors leads to synaptic current peak within ⁓0.5–1 ms (Koch, 1999). Individual postsynaptic currents decay within milliseconds, as neurotransmitters are captured by specific transporters facing the synaptic cleft, or degraded by extracellular enzymes. In contrast, both neuropeptides and neurotrophic factors slowly act in a volume of tissue, whose dimension depends on their spreading profile. This is governed by molecule size and stability, tortuosity of interstitial spaces, and binding to specific receptors, matrix proteins, or other extracellular components. Long permanence and wide-range diffusion are hence typical of peptides in CNS extracellular spaces, because of (i) mismatch between the release sites and peptide receptor distribution (Herkenham, 1987), (ii) lack of rapid degradation or reabsorption mechanisms, (iii) high peptide resistance to protease action, determined by posttranslational modifications. Classic examples are vasopressin and oxytocin, with extracellular half-lives of tens of minutes (Ludwig and Leng, 2006). Such dynamics differs from the rapid long-distance distribution through blood in endocrine signaling and the usually (although not necessarily) quicker local action of classic neurotransmitters.

In the following, we compare the physiology of orexins (neuropeptides) and neuregulins (neurotrophic factors). Despite their different biological functions, these systems display analogies in their extracellular actions, whose analysis epitomizes the experimental challenges posed by peptide neurotransmission. For summary, see Table 1 and Figure 1.

**Table 1:** Main structural and functional features of orexins and neuregulins.

Element/feature	Orexins	Neuregulins
Genes	*Hcrt*	*NRG1-NRG6*
Precursors	Prepro-orexin	Pro-NRG1-6 (membrane-bound)
Mature peptides	Orexin-A; Orexin-B	NRG1-NRG6*
Molecular weight (Da)	3,562 (Orexin-A); 2,937 (Orexin-B)	7–8,000 (mature NRG1 isoforms)
Amino acid residues	33 (Orexin-A); 28 (Orexin-B)	>60 (NRG1)
Diffusion coefficient in CNS interstices (order of magnitude estimate)**	⁓10^−6^ cm^2^ s^−1^	⁓10^−6^ cm^2^ s^−1^
Binding to ECM	Scarce	Binding to heparan-sulfate proteoglycans and other ECM components
Stability in CSF	Hours (for Orexin-A)	Steady levels of NRG1 Uncertain kinetics***
Receptors	OX_1_—OX_2_	ErbB2—ErbB4
Receptor type	G protein-coupled receptors	Tyr kinase
Receptor’s down-regulation by ligand	Slow	Very slow

Comparison of the basic structural and dynamic characteristics of orexins and neuregulins. Full treatment is given in the main text. *Each NRG type comprises isoforms (not listed in the table), because of alternative transcription and splicing. E.g., NRG1 comprises more than 30 isoforms. **No exact determinations for orexins or NRGs are reported in literature, to the best of our knowledge. Our approximate estimate follows Weiss (1996), assuming diffusion coefficients in free aqueous solution of ⁓2·10^−6^ cm^2^ s^−1^ for polypeptides of 3 to 8 kDa, at 20–25 °C. These values must be divided by 2–3, to account for the slower diffusion through CNS interstices (Syková and Nicholson, 2008). ***Steady concentrations of NRG1, in equilibrium with NRG1 bound to ECM, are detected in human brain CSF (Pankonin et al., 2009; Mouton-Liger et al., 2020). No determinations are available for the time course of NRG level in CSF, after transient local release or injection.

**Figure 1:**
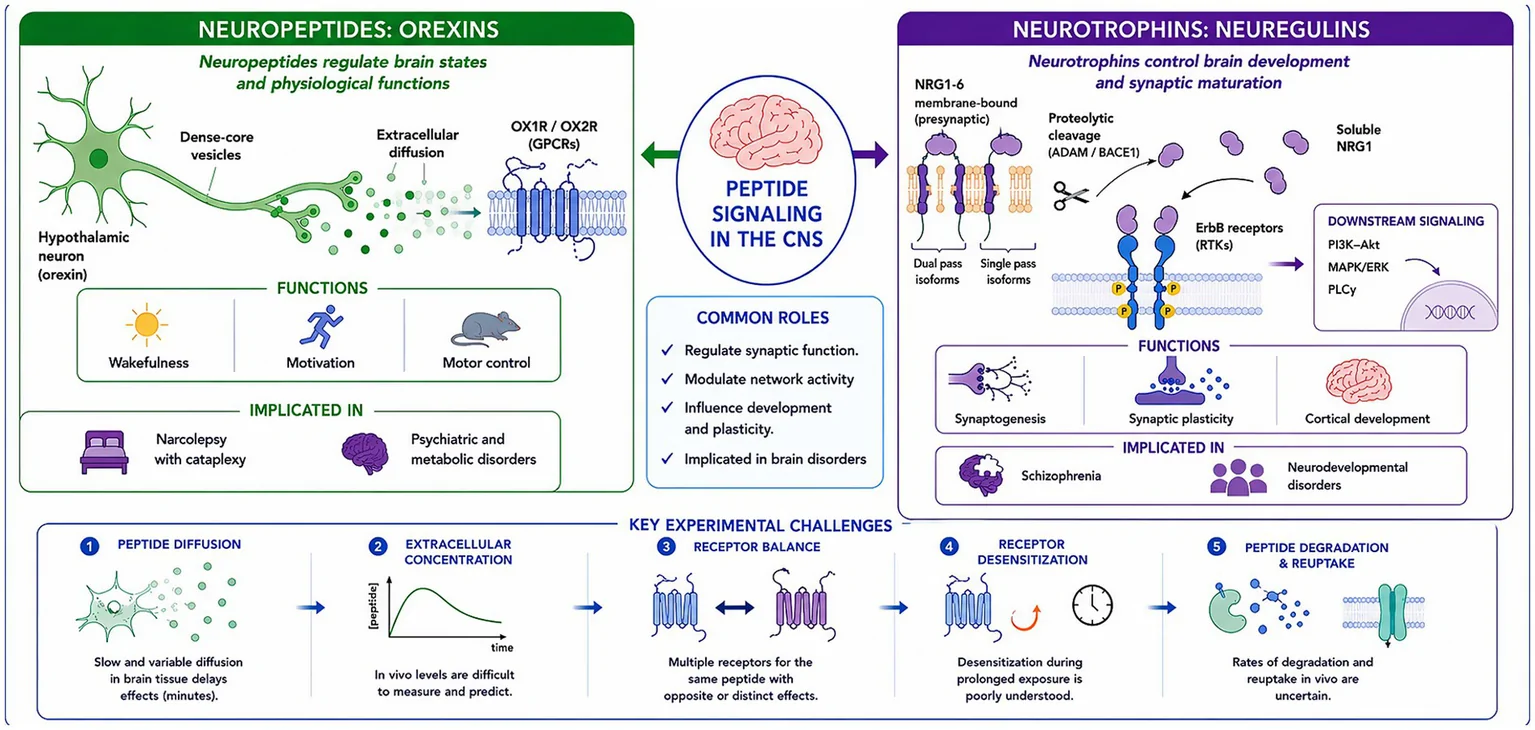
Schematic illustration of the mechanisms of action and physiological functions of orexins (left) and neuregulins (right). The bottom panel summarizes some of the main uncertainties about peptide action in the CNS.

## 2 Orexins

Orexin-A and -B (or hypocretin-1 and -2) are generated by proteolytic cleavage of prepro-orexin and secreted by neurons located in the lateral hypothalamus. Orexin-A has an N-terminal pyroglutamate residue and two intrachain disulfide bonds. Orexin-B is a linear peptide with 46% of identity with orexin-A. Both are stabilized by C-terminal amidation. Orexin-A and -B and their G-protein-coupled receptors OX_1_ and OX_2_ are highly conserved among mammals (de Lecea et al., 1998; Sakurai et al., 1998). Discharge of orexin neurons is maximal during active waking, decreases during quiet waking and is minimal during sleep, except for activity bursts during phasic rapid-eye-movements sleep (REMS; Lee et al., 2005; Mileykovskiy et al., 2005). Orexin projections are found throughout the brain, and particularly in the subcortical arousal regulatory nuclei, the neocortex, and the spinal cord (Jones, 2020). Many orexin terminals co-release glutamate (Torrealba et al., 2003; Henny et al., 2010; Qi et al., 2026). Orexin neurons integrate input about environmental and biological rhythms, body metabolism, and psychological state to set the goal-oriented activity motivated by physiological drives (Kukkonen et al., 2024).

As other ascending regulatory systems, orexin neurons project to the forebrain and the subcortical nuclei projecting therein, which considerably complicates the underlying network dynamics. For instance, orexin fibers densely innervate the dopaminergic neurons in the ventral tegmental area (VTA) as well as the medial prefrontal cortex (PFC) (Peyron et al., 1998; Fadel and Deutch, 2002). The VTA projections to the nucleus accumbens regulate reward- (or aversive-) driven motivated behaviors (Klawonn and Malenka, 2019), while the VTA-PFC pathway modulates cognition and is implicated in neuropsychiatric disorders (Boutrel et al., 2010). Hence, understanding how orexins modulate dopamine function requires to determine: (i) the distinct OX_1_ and OX_2_ roles in VTA populations; (ii) how these populations regulate mesolimbic and mesocortical dopaminergic output; (iii) the interplay of orexins and dopamine in PFC circuits. Defining the parallel effects on subcortical and cortical targets is thus essential to understand orexin pathophysiology, as exemplified by the case of narcolepsy type-1. Orexin deficit, whose causes are debated (Vassalli et al., 2026), causes excessive daytime sleepiness punctuated by sudden losses of muscle tone triggered by intense emotions (cataplexy). These manifestations may be accompanied by symptoms such as hallucinations, sleep-onset REMS events (Bassetti et al., 2019) and cognitive impairment (Harel et al., 2024). Whereas sleepiness can be broadly justified by under-stimulation of the arousal system, the link between positive emotions and loss of motor control calls into play a complex orexin-modulated network, comprising limbic areas and the medial PFC (e.g., Oishi et al., 2013). The untimely REMS events could instead reflect unbalance between hypothalamic populations, with defective orexin neurons being unable to counteract the action of REMS-stimulating melanin-concentrating hormone neurons (Naganuma et al., 2018). Interaction between orexin neurons, which stimulate wakefulness and palatable food consumption, and melanin-concentrating hormone neurons, sustaining sleep and caloric food consumption could also underlie orexin-dependent metabolic and psychiatric disorders (Bouâouda and Jha, 2023).

To settle such issues, and define the respective contributions of OX_1_ and OX_2_, complementary experimental methods must be applied to wild-type and genetically modified animals. Murine lines are available in which OX_1_, or OX_2_, or both are constitutively deleted. Conditional transgenic strains or viral vectors allow to control receptor expression in time or in selected neuronal populations (Kukkonen et al., 2024). Yet, the contribution of either receptor to local circuits must be ultimately determined with pharmacological tools. Using orexin-A or -B is not unequivocal. Orexin-B is thought to be more selective for OX_2_ (Sakurai et al., 1998), but the relative effects of the two peptides on OX_1_ and OX_2_ depend on the expression system and the activated downstream pathway (Kukkonen, 2013). To some extent, similar caveats apply to more specific synthetic agonists, such as Ala^11^, D-Leu^15^-orexin-B (Asahi et al., 2003), so that comparing several agonists and antagonists is advisable. What is worse, orexins likely act *in vivo* at concentrations lower than applied experimentally. To produce visible cell responses in brain slices, agonists must be often applied at saturating concentrations (≥100 nM; but sometimes in the μM range; Aracri et al., 2015; Ishibashi et al., 2016; Irukayama-Tomobe et al., 2017). This is likely due to low receptor expression in target neuronal populations, as smaller orexin concentrations are efficacious in cells with high receptor expression, e.g., OX_2_ in tuberomammillary histaminergic neurons (Bayer et al., 2001). Under this respect, care should be taken in distinguishing direct effects on orexin receptors expressed in the somatodendritic compartment (‘postsynaptic’; e.g., Bayer et al., 2004), from indirect effects caused by modulation of neurotransmitter release from surrounding, possibly sparse, fibers (‘presynaptic’; e.g., van den Pol et al., 1998; Lambe et al., 2005; Borgland et al., 2008; Aracri et al., 2015). Moreover, the experimental action of any peptide is delayed by diffusion from the surface to the core of tissue explants under bath perfusion. In particular, the target cell’s response to orexins often retards 1–2 min after drug application (Bayer et al., 2001; Ishibashi et al., 2016; Irukayama-Tomobe et al., 2017; Bandarabadi et al., 2024). Unfortunately, the delays caused by the perfusion system inertia are rarely specified. Nevertheless, delays of minutes are expected simply because of peptide diffusion over hundreds of μm (Table 1).

More information is available on orexin stability. Intracerebral ventricle injections of 10 nM orexin-A or -B give strong and long-lasting (hours) elevations in cerebrospinal fluid of orexin-A, but not orexin-B (Lindström et al., 2021). Orexin-A is protected from peptidase action by multiple post-translational modifications and would thus seem apt to modulate slowly changing brain state transitions. Orexin-B could shape the local circuit dynamics on a quicker time scale, in line with the heterogeneity of OX_2_ action. OX_2_ engagement is increasingly recognized to also entail direct cell inhibitory effects (Leonard and Kukkonen, 2014; Belle et al., 2014; Izawa et al., 2025; Tzanoulinou et al., 2026), which could substantially increase the signal flexibility of local circuits under simultaneous control by orexin-A and -B. How OX_1_ and OX_2_ desensitization contribute to the respective physiological roles is unclear. Although OX_2_ appears to interact more strongly with β-arrestins in cell lines, current evidence is insufficient to infer clear *in vivo* differences between OX_1_ and OX_2_ desensitization/internalization (Dalrymple et al., 2011; Kukkonen et al., 2024).

Finally, the actions of orexins must be related to the faster effects of co-released glutamate, which could explain how orexin neurons rapidly link sensory stimulation (González et al., 2016; Hassani et al., 2016) to motor output and adaptation (Karnani et al., 2020; Donegan et al., 2022).

## 3 Neuregulins (NRGs)

NRGs are neurotrophic factors related to EGF, which regulate neurodevelopment, synaptic plasticity in the mature brain, and nonneural tissue integrity (Cespedes et al., 2018). Six genes are known to date, *NRG1-NRG6*, yielding tens of variants by alternative transcription and splicing (Ledonne and Mercuri, 2019). *NRGs* encode membrane precursors (pro-NRGs) constituted by an intracellular C-terminal tail, a transmembrane domain and an extracellular EGF-like domain, followed by different N-terminal structures. We here focus on NRG1 and NRG3, the most thoroughly studied in CNS. Pro-NRG1 presents six isoforms (I-VI), bearing different N-terminal domains. In pro-NRG1 I-II and IV-V (membrane *single-pass* isoforms), the EGF-like domain is followed by an immunoglobulin (Ig)-like domain, which is absent in pro-NRG1 III and VI. Instead, in pro-NRG1 III, the EGF-like domain is followed by a second transmembrane segment adjacent to a cysteine-rich domain (Kataria et al., 2019). Such membrane *dual-pass* structure is also found in pro-NRG3 type-I, the main isoform in postnatal brain (Nadeem et al., 2025). In single-pass isoforms, NRG maturation involves proteolytic release of the extracellular N-terminal moiety (*ectodomain*). This acts in a paracrine way, as the EGF-like domain binds Tyr kinase ErbB-type receptors in target cells (Mei and Nave, 2014). Proteolytic NRG1 shedding is carried out by A Disintegrin and Metalloproteinases (ADAMs), or β-secretase −1 (BACE-1). Protease activity is regulated by cell population-specific release mechanisms, usually involving [Ca^2+^]_i_ elevation modulated by neurotrophins or glutamate, as a function of neuronal activity (Iwakura et al., 2017). Conversely, in dual pass NRGs, the EGF-like domain remains linked to the membrane-bound N-terminal domain, after proteolysis. In this case, mature NRGs act as cell–cell adhesion molecules, activating postsynaptic ErbB receptor without being released in the extracellular space (Kataria et al., 2019; Nadeem et al., 2025). On ligand binding, ErbB receptors form homo- or heterodimers. The ensuing activation of intracellular Tyr kinase domains triggers classic intracellular pathways (PI3K-Akt–mTOR, the Ras pathway, PLC-PKC), or non-canonical signals: (i) Proteolytically released intracellular ErbB4 domains can directly modulate gene expression; (ii) ErbB4 ectodomains can be shed and bind pro-NRGs, thus interfering with NRG function, or causing retrograde NRG signals; (iii) Proteolytically released pro-NRG intracellular domains regulate downstream signals and protein turnover (Mei and Nave, 2014; Ledonne and Mercuri, 2019).

NRG1/ErbB signals shape neural circuit development, by regulating cell differentiation, neurite guidance and synapse formation. Early alteration of NRG signaling can derange CNS synaptic balance, possibly causing neurologic and psychiatric diseases, including bipolar disorders and schizophrenia (Mei and Nave, 2014). The NRG/ErbB axis also operates in the adult nervous system. First, NRG1 is implicated in demyelinating diseases. Its contribution is essential in all stages of PNS myelination, although more controversial in CNS (Ziaee et al., 2026). Second, NRGs intervene in CNS trauma and ischemia. By modulating immune B and T cell populations and microglia, NRGs sustain a pro-regenerative anti-inflammatory setting that favors tissue repair and wound healing (Kataria et al., 2019). Finally, NRGs contribute to synaptic plasticity, by controlling long-term potentiation and depression in hippocampal CA3-CA1 synapses, and long-term depression in midbrain dopaminergic neurons (Ledonne et al., 2015, 2018; Ledonne and Mercuri, 2019; Huang et al., 2000). Moreover, NRGs modulate expression and function of nicotinic ACh receptors (Chang and Fischbach, 2006; Zhong et al., 2008; Zhou et al., 2018) and GABA_A_receptor (Mitchell et al., 2013), in hippocampus and neocortex. The relative contribution to permanent cognitive deficit of NRG-dependent synaptic alterations occurring during development or in mature circuits remains to be determined.

The NRG impact on CNS circuits critically depends on the spatiotemporal range within which the ligand remains biologically available in extracellular microenvironments. Like orexins, and neuropeptides in general, the released NRGs have structural features, such as the Ig-like domain, which greatly enhance stability and efficiency of interaction with ErbB receptors, with EC_50_’s in the nM range (Eto et al., 2006). Yet, NRG1 (the best understood) does not diffuse in a radial manner through the extracellular space as freely as neuropeptides. The difference depends not so much on NRG size, which only lowers the estimated diffusion coefficient by 2–3 times compared to orexins (Table 1), as on interaction with heparan-sulfate proteoglycans and other ECM components. By retaining and concentrating NRGs, the peri-synaptic environment constrains peptide mobility and signaling range (Frischknecht and Gundelfinger, 2012). Moreover, specific proteases like neuropsin can cleave the heparin-binding domain located at the NRG1 N-terminus, further modifying its interaction with ECM and biological half-life (Tamura et al., 2012). The kinetics of NRG spread is thus determined by activity-dependent release, free diffusion, capture and buffering by extracellular matrix, enzymatic processing, distribution and accessibility of ErbB receptors. The latter is potentially modulated by desensitization through clathrin-mediated NRG-ErbB internalization (Sorkin and Goh, 2009). Notably, receptor down-regulation is considerably slow for ErbB2-4 receptors. In presence of its ligand, ErbB4 decreases its half-life by 10–20%, compared to ⁓75% for ErbB1/EGFR (Baulida et al., 1996), suggesting a prolonged target cell responsivity to NRGs. Nonetheless, under sustained stimulation, receptor desensitization is reinforced by ErbB ubiquitination, whose effect is different among ErbB4 isoforms (Sundvall et al., 2008).

A supplementary regulatory layer arises from distinct subcellular localization of NRG isoforms. A major example is the modulation exerted by ErbB4 on GABAergic interneuron wiring. In rodent neocortex, ErbB4 is almost exclusively found in parvalbumin-expressing GABAergic cells, where it regulates the formation of glutamatergic synaptic input and the inhibitory synaptic output on target neurons (Fazzari et al., 2010; Ting et al., 2011; Yang et al., 2013). The relative contribution of NRG1 and NRG3 is debated. Recent work shows that, in pyramidal neurons, the respective intracellular C-terminal domains target NRG1 to the somatodendritic compartment and NRG3 to the axon terminals (Exposito-Alonso et al., 2020). Thus, dual-pass NRG3 would be enriched on glutamatergic boutons and, after proteolytic exposure of EGF-like domains, bind to ErbB4 on the GABAergic somatodendritic membrane. In general, NRG3 and the analogous dual-pass NRG1 subtypes would contribute to organize excitatory–inhibitory synaptic networks by cell-to-cell contact (Vullhorst et al., 2017; Exposito-Alonso et al., 2020). In contrast, the single-pass pro-NRG1 isoforms are primarily enriched in somatodendritic compartments of pyramidal cells. In these, activity-dependent ectodomain shedding rapidly generates soluble ligands (Shamir and Buonanno, 2010; Vullhorst et al., 2017). Hence, the effective NRG signal experienced by parvalbumin-expressing interneurons depends on whether ligand presentation originates from somatodendritic or axonal compartments of pyramidal cells, adding a further regulatory dimension to NRG–ErbB signaling and its derangement in neuropathology.

## 4 Experimental challenges and conclusions

The dynamics of peptide action in the CNS remains poorly understood. Chemical synthesis has offered increasingly specific molecules to distinguish receptor subtypes. Moreover, the availability of murine strains conditionally expressing distinct peptide receptors in specific neuronal populations is growing. Coupling these tools with focal optogenetic modulation promises to unravel complex interactions between brain regions. Still, little quantitative information is available about peptide release, diffusion, and degradation, receptor desensitization, and the balance of the effects produced by peptides and co-released neurotransmitters. Recent advances in amperometry allow to simultaneously follow the extracellular release and disappearance of enkephalins (5 aa) and catecholamines from single chromaffin cells (Denison et al., 2025), and dopamine and met-enkephalin in VTA slices (Berger et al., 2025). Such methods have been extended to neuropeptide-Y (36 aa; Alyamni et al., 2025), which promises further extension. However, in large peptides the appropriate aa targets (typically Tyr) could be unavailable or shielded. Moreover, the assay specificity should be carefully checked when multiple peptides are co-released. Nonetheless, this approach offers unique insight into the peptide release rate as a function of presynaptic activity and the action range and kinetics of neurotransmitters and neuropeptides. Alternatively, peptide distribution could be visualized after tagging with fluorescent molecules. Indeed, the spatiotemporal regulation of NRG1 shedding has been studied with NRG1 Cleavage Indicating SenSOR in cell lines and zebrafish embryos (Kamezaki et al., 2016). However, the great recent advances in live imaging techniques (Deguchi et al., 2025) have been scarcely applied to peptide neurotransmission. A supplementary technique is sampling steady peptide levels in CSF, for diagnostic purposes or to compare peptide stabilities. Liquid chromatography-mass spectrometry offers higher specificity than immunoassays. *E.g*., mass spectrometry in CSF samples (Lindström et al., 2021) confirmed the higher stability of orexin-A suggested by immunoassay (Yoshida et al., 2003), but revealed lower overall peptide concentrations. A complementary strategy to obtain physiologically meaningful results would be to attempt controlled spatiotemporal application of synthetic photoreleasable drugs mimicking the peptide action in brain slices, or in 3D cell cultures grown on microfluidic systems to model *in vivo* peptide working. Combination of the above approaches promises to advance integrative understanding of neuropeptides and neurotrophic factors in CNS.

## Data Availability

The original contributions presented in the study are included in the article/supplementary material, further inquiries can be directed to the corresponding author.

## Glossary

aa: amino acid residue
ACh: acetylcholine
ADAM: A disintegrin and metalloproteinase
Akt: protein kinase B
BACE-1: β-secretase 1
BDNF: brain-derived neurotrophic factor
[Ca^2+^]_i_: cytosolic free Ca^2+^ concentration
CSF: cerebrospinal fluid
CNS: central nervous system
EC_50_: half-effective concentration
EGF: epidermal growth factor
ECM: extracellular matrix
ErbB: EGF receptor related family
GABA: γ-aminobutyric acid
LGV: large granular vesicle
mTOR: mechanistic target of rapamycin
NGF: nerve growth factor
NRG: neuregulin
NT: neurotrophin
OX_1_: orexin receptor 1
OX_2_: orexin receptor 2
PFC: prefrontal cortex
PI3K: phosphatidylinositol 3-kinase
PKC: protein kinase C
PLC: phospholipase C
PNS: peripheral nervous system
p75NTR: p75 neurotrophin receptor
Ras: small G protein activating the MAP kinase pathway
REMS: rapid eye movement sleep
Trk: tropomyosin receptor kinase
VTA: ventral tegmental area
